# The impact of compression and confinement in tumor growth and progression: emerging concepts in cancer mechanobiology

**DOI:** 10.3389/fmats.2025.1492438

**Published:** 2025-04-08

**Authors:** Allison McKenzie Johnson, Charles Froman-Glover, Akshitkumar Mistry, Kavitha Yaddanapudi, Joseph Chen

**Affiliations:** 1 Department of Bioengineering, University of Louisville, Louisville, KY, United States; 2 Department of Medicine, University of Louisville, Louisville, KY, United States; 3 Department of Neurosurgery, University of Louisville, Louisville, KY, United States; 4 UofL Health – Brown Cancer Center, University of Louisville, Louisville, KY, United States; 5 Immuno-Oncology Program, Brown Cancer Center, Department of Medicine, University of Louisville, Louisville, KY, United States; 6 Division of Immunotherapy, Department of Surgery, University of Louisville, Louisville, KY, United States

**Keywords:** mechanobiology, tumor microenvironment, bioengineered platforms, microfluidics, solid stress, cancer

## Abstract

Cancer is one of the deadliest diseases despite aggressive therapeutics. This is due in part to the evolving tumor microenvironment (TME), which provide tumor supportive cues that promote tumor adaptation and progression. Emerging studies highlight the significant role of the biophysical characteristics in the TME in modulating all aspects of cancer aggressive and spread. With the advance of bioengineering platforms, deeper investigations into the impact of these biophysical features on cancer progression are being conducted with a growing appreciation of the intratumoral compression that underlie many of the biophysical changes. Intratumoral compression emerges early in tumor development and increases in magnitude as the tumor rapidly expands against itself and its surrounding tissue. This stress has effects on both the cancer cells and biophysical aspects of the TME, including hypoxia, shear stress, extracellular matrix (ECM) remodeling, and substrate stiffness. This creates a physically dense, pro-malignant environment that can both promote metastatic phenotypes and spread but also present biophysical barriers for immune cell infiltration. This review will analyze the effect of compressive stress on the TME, cancer cells, and on confined migration of cancer and immune populations.

## Introduction

Cancer is one of the leading causes of death worldwide, accounting for 9.7 million deaths alone in 2023 (Siegel et al., 2023). With the increase in life expectancy and technological advances in diagnostics, the number of cancer diagnosis is predicted to increase in the coming years (Weir et al., 2021). In the past 50 years, tremendous advances have been made in cancer diagnosis and treatment, resulting in ~20% increase in the 5-year survival rates of cancer patients since the 1970s (Miller et al., 2022). Established therapeutic strategies include combinations of surgical recission, chemotherapy, and radiation therapy. New and more targeted strategies have emerged with the application of monoclonal antibodies and immunotherapies. Commonly dysregulated pathways, including PI3K/Akt, JAK/STAT, Hedgehog, Notch, and Wnt/β-catenin have all been the focus of targeted therapies in cancer cells and cancer stem cells, showing promise in preclinical models (Liu and Wang, 2024; Sloan et al., 2023; Li et al., 2015). For example, napabucasin, a STAT3 inhibitor, and GDC-0449, a hedgehog pathway inhibitor, were shown to prolong survival in clinical trials (Sloan et al., 2023; Li et al., 2015). Within the microenvironment, targeted blockade of vascular endothelial growth factor (VEGF), such as bevacizumab, signaling to interrupt angiogenesis and deplete the tumor nutrient supply has shown promise *in vitro* and has led to FDA approval in several cancer types (Sasich and Sukkari, 2012). Efforts to leverage the immune system to target and attack cancer has shown tremendous promise in several blood cancers and some solid tumors (Kciuk et al., 2023). Immune checkpoint inhibitors, T-cell transfer therapy, and the use of monoclonal antibodies all enhance the immune system’s ability to recognize and attack tumor cells (Liu et al., 2022). There are several excellent reviews that survey these next-generation strategies (Hamdan and Cerullo, 2023; Patel et al., 2024).

Although cancer treatments have advanced significantly, there is still a high mortality rate, especially in the most malignant and aggressive solid tumors. In addition, patients can also become disabled through disease progression and treatments, which further diminish quality of life. In 2019, there was an estimated 250 million disability-adjusted life years (DALY) from cancer alone, a 16% increase since 2010 (Collaboration et al., 2022). Thus, there is still a critical need for the development of additional therapeutic avenues to combat this deadly disease. Tumor cells exist in a complex ecosystem called the tumor microenvironment (TME), which plays a tumor supportive role, enabling tumor adaptation and evolution to evade treatment. Within the TME, a heterogeneous array of biomolecular and biophysical signals from various anatomic, necrotic, and hypoxic niches regulates tumor behavior (Baxter et al., 2023; Wolf et al., 2019). Recent reports have begun to shed light on the underappreciated impact of the biophysical cues embedded in these niches (Engler et al., 2006; Lo et al., 2000; Polacheck et al., 2014; Mahaffey et al., 2024; Teer et al., 2024; Chen and Kumar, 2017). Changes in tumor stiffness, shear stress, interstitial pressure, and solid stress have all been documented during tumor growth and evolution (Emon et al., 2018). Each of these signals can influence cancer cell proliferation, migration, and invasion independent of biomolecular stimulus, highlighting their significant role in facilitating tumor aggressiveness. Investigations into the mechanism underlying these pro-malignant biophysical signals can lead to the identification of a novel suite of TME targeting therapies. In fact, several reports have described successful pharmacological approaches to combat cancer progression by disrupting different aspects of the mechanotransductive network, including cytoskeletal elements (actin, microtubules), cytoskeletal regulators (RhoGTPase proteins), and proteins involved in contractility (myosin-II) (Di et al., 2023; Xin et al., 2023). Continued efforts to elucidate these mechanotransductive mechanisms will help push the envelope of new therapeutic avenues for cancer treatment.

Advances in our understanding of the pro-malignant effect of biophysical cues largely depends on the development of adequate bioengineered platforms capable of recapitulating these mechanical cues (Wolf et al., 2019; Chen et al., 2021). Thus, many of the important insights gained in cancer mechanobiology have been established in 2D models leveraging biomaterials and microfluidics. However, as more complex tools emerge, deeper investigations of the role of the biophysical TME in the 3D context are being considered. One emergent biophysical concept relevant in the 3D context is the recognition that cancer cells exist in tight, confined environments during tumor growth and spread (Chauhan et al., 2014). Cancer growth imposes a sustained 3D intratumoral compressive stress upon cells that have been shown to enhance migratory capacity in breast cancer (Tse et al., 2012). Further, cells leaving the primary tumor in the metastatic cascade must squeeze through a dense 3D matrix, intravasate and extravasate vasculature to successfully form secondary tumors (Vasilaki et al., 2021). Studies have revealed that aggressive cancer cells can more efficiently traverse this landscape when compared to less aggressive cancer cells and that the ability of cells to limit DNA damage is central to this process (Denais et al., 2016). Early reports have shown the importance this biophysical axis on cancer progression and spread; however, many of these mechanisms are still poorly understood.

In this review, we focus our discussion on the impact of compression and confinement on tumor progression and spread. We describe the current understanding of these biophysical signals on tumor proliferation, phenotype, and invasion, as well as their role in regulating tumor supportive and anti-tumor immune populations. Lastly, we survey the novel bioengineered platforms developed to interrogate these interactions and offer thoughts on the future steps and clinical opportunities of this area of investigation.

## The emergence of the biophysical TME

The biophysical TME develops through a cascade of processes that involve multiple cell types and occur across various time points. During early stages of tumor formation, cancer associated fibroblasts (CAFs) are recruited to the tumor site, encapsulating the tumor (Bertillot et al., 2024a). These cells and other stromal populations rearrange the matrix by degrading and secreting more extracellular matrix (ECM) proteins and increasing the cross-linking between ECM proteins, altering the tumor landscape (Belhabib et al., 2021). Cancer cells themselves also contribute to the stiffening in the TME through the excretion of metalloproteinases and secretion of ECM proteins (Lu et al., 2011). Many of these physical changes generate a feed-forward loop, promoting tissue remodeling processes that further change the biophysical properties. The abundance of crosslinked ECM elevates the rigidity of the surrounding environment and imparts pro-malignant signals to the tumor cells (Yayan et al., 2024). As the tumor expands, it also encounters resistance from the surrounding tissue. This resistance grows as the tumor expands, imparting elevated compressive stress on the tumor. This rapid tumor expansion also creates tensile stress across the perimeter of the tumor, which creates cellular strain that can stretch open ion gated channels on cell membranes, allowing for the influx of ions and proteins to promote pro-tumorigenic responses (Otero-Sobrino et al., 2023). The continued expansion of the tumor and secretion of elevated ECM create heterogeneous stress and strain distributions across the tumor, collapsing tumor vasculature and creating zones of necrosis and hypoxia (Chen et al., 2023). The late stage TME is rich with secreted factors, zones of oxygenation, and importantly a dense ultrastructure that becomes a barrier for anti-tumor cells (Mahaffey et al., 2024).

**FIGURE 1:**
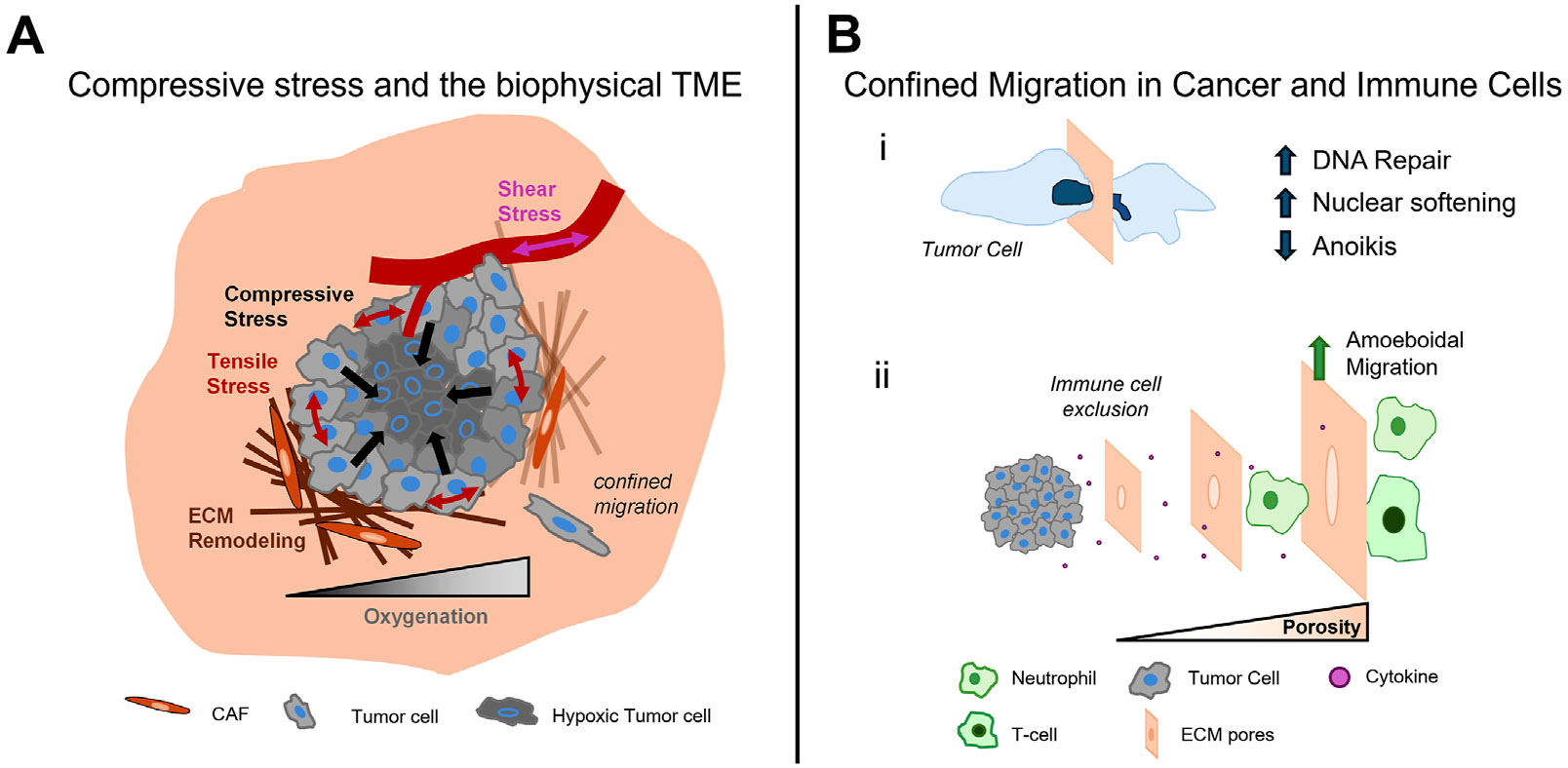
The impact of compression and confinement in cancer progression. The growth of tumors in confined environments leads to the increase of compressive stress within the tumor and heightens tensile stress at the tumor periphery. The evolution of these stresses disrupt interstitial fluid flow and oxygen availability, shear stress in tumor vasculature, and the activation of ECM remodeling, which further alters the tumor landscape **(A)**. In the dense TME, cells experience confined migration, which cancer cells overcome through increased DNA repair, increased nuclear compliance, and a resistance to anoikis (i). The dense TME also affects immune cell infiltration as tumor pore size decreases. Neutrophils and T cells adopt amoeboidal modes of migration with neutrophils showing high efficiency in squeezing through smaller pores; however, as pore size decreases immune cell infiltration is restricted (ii) **(B)**.

Cancer cells interpret the mechanical changes in this environment through mechanotransductive pathways (Chin et al., 2016). Mechanotransduction is a tightly regulated process involving mechanosensitive receptors and proteins that signal through the interconnected cytoskeleton to induce transcriptional changes to the cell. Mechanical signals are sensed through a variety of mechanosensitive receptors including cell-ECM receptors (integrins, CD44), cell-cell junctions (cadherins), mechanically gated ion channels (piezos), and other transmembrane proteins (glycocalyx) (Vining and Mooney, 2017). Once sensed, the mechanical signal is primarily transduced through cytoskeletal elements including the actin filaments with microtubules and intermediate filaments playing important roles as well. The cumulative effect of mechanical signals leads to distinct changes in gene expression, protein cascades, and even cell fate (Li and Wang, 2020). Within the biophysical TME, cancer cells are continually under these pro-malignant mechanical signals, which facilitate and support tumor evolution and growth. Additionally, cells are now encased in dense microenvironments that force cells to adopt specific modes of migration to navigate this increasingly confined environment. This selects for privileged populations of cancer cells to metastasize but also restricts anti-tumor immune cells from infiltrating into the tumor mass. Thus, aberrant biophysical environments contribute significantly to cancer progression and spread and present novel pharmacological targets for cancer therapy. The biophysical features of the TME are complex and interrelated, and we detail its interconnected nature starting with the growth of compressive stress in early stages of tumor development and its eventual role in promoting changes in shear stress, tension, and tissue stiffness (Figure 1A).

### The impact of compression on tumor progression

#### Compressive stress contributions to TME

Solid stresses accumulate inside the tumor due to the rapid proliferation of cancer cells, which encounters resistance from the surrounding tissue as it expands. The magnitude of compressive stress increases as the tumor grows, providing a dynamic biophysical input that shapes the tumor microenvironment (Nia et al., 2016; Stylianopoulos et al., 2013). The emergence and growth of this compressive stress affects all aspects of the development of the biophysical TME, fostering a tumor supportive environment that underlies many facets of cancer progression (Table 1). This section will review the effects of compressive stress on several aspects of the TME and their contribution to promoting malignancy.

#### Hypoxia

Compressive stress in the TME applies pressure on the lymphatic and vascular systems, which can collapse the blood vessels and alter the fluid flow across the TME (Northcott et al., 2018). This compressive environment can lead to compensatory activation of VEGFA, a cytokine important in angiogenesis, in the CAF population, which creates a chemotactic gradient encouraging angiogenesis to the increasingly necrotic core as the tumor develops (Kim et al., 2017b). However, tumor vasculature exhibits leakiness and reduces the overall nutrient deposition into highly compressed areas of the tumor, increasing hypoxia and promoting malignancy. Solid stress also reduces the flow of nutrients in tumor spheres. A 3D microfluidic spheroid system showed that the addition of compressive stress on an *in-vitro* tumor spheroid lead to the formation of a necrotic core and increased proliferation in the outer regions of the tumor spheroid (Alessandri et al., 2013). The alterations of oxygenation and nutrient supply lead to changes in cancer cell metabolism, cancer cell phenotype, therapy resistance, and immune cell activity. The hypoxic environment leads to the shift of oxidative phosphorylation to anaerobic glycolysis, decreasing the reliance on available oxygen for cell proliferation (Vaupel et al., 2001; Koukourakis et al., 2003). This metabolic shift is also seen to promote an increase in invasion through a mesenchymal shift (Yang et al., 2020), and intermediates play a role in activating ZEB1, a mesenchymal transcription factor (Liu et al., 2021). A hypoxic environment can also influence the therapeutic resistance of cancer, as cancer cells in hypoxic environments are significantly more likely to survive radiotherapy compared to a normoxia condition (Carlson et al., 2006). Additionally, hypoxic conditions contribute to the lack of available oxygen needed for aerobic glycolysis for T-cell function (Chang et al., 2015), in which regulatory T-cells flourish further promoting an immunosuppressive response (Angelin et al., 2017). Cytotoxic T-cells and Natural Killer cells in the hypoxic environment exhibit a reduction in tumor suppression due to the increase of lactate and increasingly acidic environment (Brand et al., 2016).

#### Shear stress

Due to collapsed vessels and altered shear stress gradient, the flow across tumor increases, thereby increasing the metastatic potential of tumor cells, specifically in prostate (Lee et al., 2017) and breast cancer lines (Qazi et al., 2013; Yang et al., 2016; Huang et al., 2018a; Mitchell and King, 2013). Shear stress can be described by τ = η*(du/dy), where η is the dynamic viscosity of the fluid and du/dy is the velocity gradient perpendicular to the flow. Changes in shear stress are sensed through the deflection of transmembrane proteins including the glycocalyx and primary cilia and also mechanically sensitive ion channels and cell-cell adhesion proteins (Espina et al., 2023). Many of these pathways lead to alterations in MAPK and PI3K/Akt proteins, which support cell survival and a resistance to apoptosis (Tzima et al., 2005). Importantly, shear stress can also directly influence cell migration and metastatic potential to resist high shear stresses and promote survival in the circulatory system. Shear stress has been shown to induce cell polarization through cytoskeletal reorganization to promote rheotaxis towards the flow origin (Polacheck et al., 2014). Increases in shear stress have also been shown to facilitate a resistance to anoikis, an apoptotic process activated when cells lose attachment to the ECM, and an increase in proliferation rates, which help cells successfully metastasize (Chivukula et al., 2015). The increase in magnitude of shear stress can also create shear stress resistance in cancer cells, allowing them to withstand high shear stresses like those seen in blood vessels during metastasis (Huang et al., 2018b). Increased exposure to shear stress is also shown to alter metabolic profiles, by changing ATP production in metastatic breast cancer (Park et al., 2022). Overall, changes in shear stress helps facilitate cancer malignancy and survival by activating cell survival pathways, altering metabolic programs, and enhancing cell migration and metastatic potential.

#### Tensile stress

Tensile stress can also emerge due to growth-induced stresses during tumor growth. As compression elevates within the tumor, the tumor periphery is stretched, leading to increased tension around the tumor perimeter. Tensile stress is quantified via σ = F/A, where F is the applied force across the cell membrane and A is the cross-sectional area of the cell membrane (Charras et al., 2018). Interestingly, this alteration in tension leads to ECM dependent changes that can facilitate tumor spread. For example, the increase of strain on the ECM reduces the elasticity of the fibers, decreasing the contractility force needed for cell movement, which promotes cellular invasion (Paszek et al., 2005). CAFs also line the ECM fibers tangent to the tumor itself, creating a radial tension in which applies an increased solid stress that the tumor has to work against during expansion (Conklin et al., 2011). With the elevated tensile stress around the tumor, cells also experience change to the intra and inter-cellar tension, which activates a host of mechanotransductive pathways that are involved in tumor progression (Northcott et al., 2018). Increased strain induces sustained extension of the cytoskeletal network, which affects actomyosin contractility, cytoskeletal architecture, and even changes to the nuclear lamina through the linker of nucleoskeleton and cytoskeleton (LINC) complex (Khilan et al., 2021). There is a reciprocal relationship between cell contractility and applied tension, which has been difficult to detangle; however, several reports provide insights to this interaction. Activation of cytoskeletal regulators RhoA/Rap1 has been shown to regulate integrin signaling, FAK and ERK1/2 phosphorylation, and activate glioblastoma proliferation (Sayyah et al., 2014). In tumor associated fibroblasts, a feedforward loop involving mesenchymal pathways JAK1/STAT3 and ROCK-mediated contractility has been identified, which leads to ECM remodeling (Sanz-Moreno et al., 2011). Similarly, in pancreatic ductal carcinoma, JAK/STAT3 also activates ROCK1, which leads to increased contractility, ECM remodeling, focal adhesion maturation and increased tumor progression and aggression (Laklai et al., 2016). Tensile stress also leads to activation of cell ECM receptors, such as integrins, which are stretched by the expanded ECM (Chen et al., 2017). Tension activates downstream integrin signaling, activating FAK and Src family kinases, which can lead to the activation of YAP/TAZ and myocardin-related transcription factors (MRTFs), which have been associated with proliferation and cancer metastasis (Sun et al., 2016). Sustained stretch can also open mechanically gated ion channels, PIEZO and Tmc, increasing ion exchange and promoting proliferation and migration (Karska et al., 2023). Lastly, increased strain elevates the intercellular tension that is distributed by cell-cell junctions, including the family of cadherins. Cadherins link cells together through extracellular domains and extend intracellularly to connect to the cytoskeleton through a complex, which includes alpha-catenin, beta-catenin, p120 catenin, and vinculin. Stretch induced cadherin mechanotransduction is still an active area of investigation, but early reports indicate an activation of Hippo signaling, which increases cell proliferation (Duszyc and Viasnoff, 2018). Further, the cadherin complex can signal through p120 catenin to regulate RhoGTPase proteins and increase migration potential (Schackmann et al., 2013). Many cancer cells also exhibit changes in their cadherin expression with a preference towards mesenchymal cadherins (N-cadherin, OB-cadherin), which may alter the response to sustained stretch (Kaszak et al., 2020). Thus, sustained tensile stress across the tumor can lead to increases in cell proliferation, phenotype, and migration potential, activating pathways through intra and intercellular mechanotransduction.

#### ECM remodeling and substrate stiffness

Intratumoral compressive stress plays an important role in the remodeling of the ECM, which alters the mechanical characteristics of the TME. Alterations in the secretion of ECM proteins from CAFs and tumor cells as well as ECM modifications and digestion of the ECM through matrix-modifying (lysyl oxidases) and matrixdegrading enzymes (MMPs) underlie many of these changes (Winkler et al., 2020). For example, applied compressive stress has been shown to alter CAF metabolism, increasing glycolysis and contributing to more active CAF based ECM restructuring (Kim et al., 2019). Compression specifically upregulated PFKFB3, which correlated with increased epithelial to mesenchymal transition (EMT) related genes (TWIST1, SNAI1, ZEB1, ZEB2, CDH1, CDH2, and MMP2) and ECM secretion (COL1A1, COL3A1, HAS2 and HAS3) (Kim et al., 2019). Remodeling of ECM interactions reveal that compression in the ECM contributes the collapse of ECM fibers, which in turn elevates ECM secretion from stromal cells and fiber densification at regions of fiber collapse (Kalaitzidou et al., 2024). Interestingly, fiber densification in these regions increase the migration of breast cancer cells regardless of substrate stiffness, suggesting that the density of cell-ECM interactions is an important regulator of malignancy (Berger et al., 2017). In addition to ECM changes in within the tumor, ECM remodeling of collagen networks is also seen at the periphery of the tumor, leading to fibers alignment radially around the tumor (Pirentis et al., 2015). These studies reveal that compressive stress can activate CAF ECM secretion, contribute to ECM fiber collapse and subsequent densification and organization, which promote cancer cell migration and invasion.

The alterations of ECM secretion and densification often lead to the increase of mechanical rigidity of the biophysical TME (Mahaffey et al., 2024; Ghasemi et al., 2020; Miroshnikova et al., 2016; Miyazawa et al., 2018; Shi et al., 2015; Mueller and Sandrin, 2010). Through mechanical probing via AFM, increases in substrate rigidity has been well documented for a variety of cancers (Magazzù and Marcuello, 2023; Massey et al., 2024). Substrate stiffness is directly tied to cancer progression and is regulated by CAF and tumor cells in most solid tumors. CAFs can secrete various soluble signals including growth factors, cytokines, and hormones which promote proliferation and invasion of the cancer cells themselves (Wright et al., 2023). This creates a positive feedback loop in which more CAFs are recruited as the tumor grows, altering more of the ECM and providing more soluble signals to promote cancer progression. Similarly, tumor cells can recruit and activate stromal cells such as CAFs into the tumor microenvironment through secretion of pro-fibrotic growth factors such as TGF-α, TGF- β, FGF-2, and EGF (Heneberg, 2016). This collectively promotes CAF recruitment and activity, leading to ECM secretion and matrix stiffening. Tumor cells themselves participate in ECM remodeling in a variety of cancers through increased expression of collagens, hyaluronic acid, and matrix modifying enzymes such as lysyl oxidases (Poltavets et al., 2018; Kai et al., 2019). Increases in stiffness also increases the cells proliferation, migration, and invasion in many cancer types (Xu X. et al., 2021; Rice et al., 2017). Matrix stiffness signals are sensed through cell-ECM receptors such as integrins, which are linked through adaptor proteins to the cytoskeleton (Sun et al., 2016). Thus, matrix rigidity dramatically affects cytoskeletal architecture, contractility, and focal adhesion assembly, essential components of cellular motility. On softer substrates, where cytoskeletal tension and contractility is low, cells struggle to form front-rear polarity, making locomotion difficult (Doss et al., 2020). However, cells on stiffer substrates, activate the cytoskeletal machinery to promote cell polarity and spread, indicating better adhesion to the ECM and potential for migration (Janmey et al., 2020; Tang et al., 2022). Mature focal adhesions also are formed on stiffer substrates and can increase contractility, through enhanced ATP localization to the actin filaments and activation of Rho/Rock signaling (Sun et al., 2010). Additionally, stiffness dependent activation of integrin signaling can lead to phenotypic changes through activation of the EMT regulatory network. This is known to influence therapy resistance, increased invasiveness, and even changes in cell compliance. Interestingly, tumor stiffness can also influence the ability of cells to displace the surrounding tissue, with more infiltration in tumors that are 1.5 times as stiff as the surrounding tissue as revealed by computational modeling (Voutouri et al., 2014).

The growth of compressive stress within the TME contributes to the dynamic development of biophysical alterations that creates the conditions for cancer cells to become more malignant over time. Further investigations into the emergence of the biophysical TME may lead to new avenues to disrupt its development and slow down its impact on tumor malignancy.

#### Compressive stress on cancer cell phenotype, immunosuppression, and chemotherapy resistance

In addition to its role in shaping the biophysical TME, compressive stress also directly confers a mechanical signal to cancer cells, activating compensatory mechanisms including increases in cortical actomyosin tension, increased vimentin intermediate filament networks, and changes in the microtubule complex (Brangwynne et al., 2006; Pogoda et al., 2022; He et al., 2018). Importantly, significant compression leads to dramatic deformations of the nucleus, which activates nuclear mechanotransductive pathways leading to alterations in transcription factor transport and modulation of gene expression.

Recent studies suggest that the nuclear mechanotransduction plays an important role in the modulation of the malignant phenotype and can be directly activated by the application of compressive stress, which has been shown to increase nuclear size and reduce circularity. Upon compression, many cytoskeletal proteins coordinate to resist the load. A significant player in responding to compression is postulated to be vimentin, with the loss of vimentin networks surrounding the nucleus increasing the deformation of the nuclei compared to those with intact vimentin networks (Pogoda et al., 2022). Microtubules also play an important role in bearing compressive loads, and rely on an interplay between the surrounding elastic cytoskeleton to do so efficiently (Brangwynne et al., 2006). Upon compression, nuclear flattening alone is sufficient to increase proliferation in a non-actin-myosin mechanotransductive manner. With the addition of compressive stress through an agarose disc, blebbistatin treated cells showed an increase in transcription factors (AP1, TEAD) that increase cellular proliferation compared to the non-compressed blebbistatin treated condition (Aureille et al., 2019). Compression dependent deformation of the nucleus is also tied to an upregulation of Piezo1, a nuclear membrane pore known to open due to tensile stretching, increasing protein localization and ion transport into the nucleus (Luo et al., 2022). Compression induced signaling also promotes invasion through an upregulation of downstream Src signaling, promoting the development of lamellipodia (Luo et al., 2022). Piezo1 upregulation is shown to be a marker of worse prognosis in both breast cancer (Xu H. et al., 2021) and gliomas (Zhou et al., 2020), indicating the higher presence of these cell surface receptors plays a role in the malignancy in these cancer types.

Compressive stress on the cell membrane can also allow for intracellular signaling across the membrane and the cytoskeleton, promoting mesenchymal signaling. Applied compression can reorganize the cytoskeleton through integrin-FAK signaling (Chen et al., 2018) to resist the compression (Greene et al., 2009) and reduce the amount of RhoA present, mediating cortical tension (He et al., 2018). Cellular cortical tension is also shown to be directly altered by the application of compressive stress. This cortical tension reduction may contribute to the cellular softening seen in early stages of metastasis, specifically in the EMT process (Hosseini et al., 2020), indicating that compressive stress may play a role in promoting the shift from an epithelial state to a mesenchymal state. Higher cortical tension in plasma membranes is seen in epithelial cells compared to mesenchymal cells, and the disruption of RhoA and ERM reduced the cortical tension, promoting a more mesenchymal phenotype (Tsujita et al., 2021). The cytoskeleton also plays a role in the mechanosensing of compressive stress, primarily by promoting the transmission of the stress to the nucleus via linker of the nucleoskeleton and the cytoskeleton (LINC) complex (Kirby and Lammerding, 2018). This mechanotransductive axis has recently been associated with YAP/TAZ mechanobiology and its role in facilitating cancer malignancy is an active area of investigation (Koushki et al., 2023).

The sustained alterations in the biophysical TME are also a regulator of immune cell function. Compressive stress in lymph-node metastasis decreased the lymphocyte presence, which was increased by excision of the tumor relieving the compressive stress (Jones et al., 2021). The aligned fibrous network promoted by the application of compressive stress also decreased the motility of T-cells, impeding the ability of tumor invasion (Kaur et al., 2019). Mechanical strain on breast cancer cells promoted the upregulation of exosomes, which spurs Myeloid derived suppressor cell recruitment and activation, reducing the effectiveness of T-cells (Wang et al., 2020). Additionally, increases in substrate stiffness promotes the release of pro-inflammatory cytokines from macrophages (Meli et al., 2020). While the direct effect of compressive stress in the TME on immune cells has yet to be fully established, the indirect effects suggest compressive stress plays a role in promoting immune evasion through the ECM and through a malignant shift.

Compressive stress has also shown to be involved in chemotherapy resistance, largely through the activation of pathways to impair proliferation (Kalli et al., 2023). For example, a computational model found that elevated compression developed in a tumor sphere growing in a confined agarose matrix exhibited reduced proliferation and resistance to gemcitabine, a chemotherapy drug that taregets proliferating cells (Rizzuti et al., 2020). The application of compressive stress on mechanically responsive breast and pancreatic cancer cells reduced proliferation through the overexpression of PI3K-isoforms, increasing therapeutic resistance (Meli et al., 2020; Di-Luoffo et al., 2025). Lastly, ovarian cancer cells under 3D compression showed an increase the chemotherapy resistance in a CDC42- dependent manner (Novak et al., 2020).

As the knowledge of the biophysical landscape of the TME increases, novel perspectives on the development and spread of cancer can lead to better therapeutics. Understanding the underlying mechanisms by which cancer can withstand high compressive forces will lead to therapeutics that interrupt or prohibit this process.

### Confined migration in cancer invasion and immune cell infiltration

In the increasingly densified TME that has been reshaped during tumor progression, cancer cells now exist in a highly confined microenvironment (Figure 1B). This new environment presents biophysical challenges to the cancer cells that desire to spread and leave the primary tumor as well as anti-tumor immune cells that arrive to facilitate tumor clearance (Henke et al., 2020). The process of cancer invasion from the primary tumor requires efficient confined migration through the dense and restrictive environment in the TME (Emon et al., 2018). Successful metastasis requires tumor cells to navigate this confined environment, which includes the tissue parenchyma, vasculature for dissemination to distant organs of the body, and parenchyma of foreign organs that exhibit unique ECM profiles. There are several biochemical and biophysical strategies that cells employ to survive the traumatic journey such as nuclear softening, and modulation of cadherin family of proteins responsible for cell-to-cell congruency (Nava et al., 2020). In turn anti-tumorigenic immune cells must also be adapt to the confined nature of the ECM so they can migrate to cancer cells and carry out their respective functions. This section of the review will highlight main biophysical cues throughout the metastatic cascade and the associated mechanisms that cancer and immune cells utilize to cope with the stresses at play within the TME and associated ECM (Majidpoor and Mortezaee, 2021).

#### Confined migration in primary tumor escape

An important consideration in primary tumor escape is the physical stress that is exerted on the escaping tumor cell as it spreads through dense tumor tissue, which possess pores or tracks that are much smaller than the diameter of a cell (Mahaffey et al., 2024; Saif et al., 2023). In such environments, cell invasion requires dramatic deformation of the nucleus, which can trigger DNA damage and mutations and importantly can activate proinvasive pathways that promote aggressive infiltration and spread (Denais et al., 2016; Kirby and Lammerding, 2018). When cells squeeze through tight spaces, nuclear envelope integrity has been shown to be disrupted leading to DNA damage and nuclear fragmentation (Denais et al., 2016). Cells that have undergone confined migration may exhibit genomic instability that can increase the likelihood of mutations and contribute to cancer progression (Fan et al., 2023). Interestingly, cells that experience repeated nuclear deformations have been shown to upregulate oncogenic pathways, including Ras/MAPK that increase the invasive potential of cancer cells (Rudzka et al., 2019). However, excessive nuclear deformation and a loss of nuclear envelope integrity can also lead to cell death; thus, only select cancer cells successfully traverse this challenging terrain. Studies have begun to unravel mechanisms that cancer cells exhibit to efficiently migrate through confined environments. For certain cancer cells, nuclear envelope repair mechanisms are elevated in an ESCRT III dependent process, limiting DNA damage and cell death (Denais et al., 2016). A recent study has also identified a mechanism by which cells resist mechanical stress by softening the nucleus through calcium dependent mechano-adaptive responses that leads to a loss of H3K9me3-marked heterochromatin and chromatin architecture (Anlaş and Nelson, 2018; Nava et al., 2020). Similarly, low lamin A levels, a nuclear envelope protein associated with nuclear stiffness, significantly correlated with decreased patient survival in breast cancer (Bell et al., 2022). These studies identify nuclear compliance as a hallmark of metastatic cancer cells. The regulation of the cytoskeleton is also critically important for efficient confined migration (Chen et al., 2020). Mechanical confinement can also trigger cell polarization and polymerization of formin based linear actin cables that enable rapid contraction and infiltration (Monzo et al., 2016). Cytoskeletal changes also underlie transitions from mesenchymal migration to amoeboidal migration. Amoeboidal migration depends less on ECM degradation of the parenchyma, and more on squeezing through the existing ECM, leading to rapid invasion through confined environments. This phenotype has been shown to be dependent on the actomyosin cortex for rapid contractility to create blebs that act to propel the cell forward. NADPH oxidase NOX4 is a metabolite involved in this transition from mesenchymal migration to amoeboidal migration and has shown potential as a pharmacological target to reduce cancer spread (Crosas-Molist et al., 2017).

#### Confined migration in intravasation and extravasation

Intravasation is a critical step in the cancer metastatic pathway, where cancer cells breach the endothelial barrier to enter the bloodstream or lymphatic system. This process is tightly regulated and represents a significant barrier to metastasis because it requires cancer cells to overcome several challenges, including adhesion to the endothelium, degradation of the basement membrane, and interaction with immune cells (Majidpoor and Mortezaee, 2021). Confinement within the primary tumor microenvironment can initially restrict cancer cell movement, but once cells become invasive, they gain the ability to navigate through tight spaces and adapt to various mechanical stresses. This adaptation is crucial for successful intravasation, as cancer cells must deform and squeeze through small gaps in the vessel walls through mechanisms similar to those described above. Once tumor intravasate into the vasculature, they become circulating tumor cells (CTCs) and are no longer supported by bulk tumor mass. In this new environment, cells are subject to a type of apoptosis called anoikis, which occurs when cells lose attachment to the ECM. Interestingly, in metastatic breast cancer cells, the processes of squeezing through confined environments generated a resistance to anoikis that sustained cell viability of CTCs (Fanfone et al., 2022). These mechanically conditioned cells also displayed increased migration potential and a surprising evasion of natural killer cell surveilance (Adeshakin et al., 2021). Thus, confined migration during intravasation and extravasation increase the metastatic potential of cancer cells by maintaining cell viability, increasing cell migration, and evading immune cell activity.

#### Confined migration and immunosuppression

A major problem with treatment of primary tumors is lack of a sufficient immune response. There are significant biophysical obstacles that immune cells must surpass to reach the tumor cells and carry out their functions. ECM composition and organization with the TME have been shown to possess pore sizes within the micron and submicron ranges, creating physical barriers that immune size must squeeze through to get to the cancer cells (Teer et al., 2024). Immune cells are guided into the tumor via chemokines and overcome physical constraints through a combination of matrix degrading enzymes and actin-based activity (Vesperini et al., 2021). Interestingly, different immune cells exhibit distinct physical and mechanical characteristics (i.e., size and deformability), which influence the way that they interact with the TME. Neutrophils appear to migrate more quickly through small pores and have much softer nuclei, compared to other immune cells (Rowat et al., 2013). Immature dendritic cells (DCs) generate a WAVE and Arp2/3 dependent actin cage, which breaks down the nuclear lamina, allowing for increased deformation of the nucleus and successful confined migration (Thiam et al., 2016). Mature DCs and T cells appear to lean on formin based mechanisms and not Arp2/3, which are involved in the formation of aligned actin structures (cables) and not branched networks (Leithner et al., 2016; Moreau et al., 2015). Importantly, myosin II, an actin-based motor protein important in contractility, is essential for DC and T cell infiltration (Soriano et al., 2011; Lämmermann et al., 2008). T cells also rely on small and dynamic focal adhesions to pull themselves through confined spaces (Caillier et al., 2024). Although some immune cells can utilize proteases to dig through the dense TME, *in vivo* studies are indicating that many immune cells bypass ECM degradation mechanisms for amoeboidal modes of invasion, which do not require specific cell-ECM receptors (Moreau et al., 2018). Many of these mechanisms are intact up until cells encounter very small pores (<1 μm), where more force is required to squeeze through. Recent studies evaluating chromatin architecture is shedding light on how cells may overcome these limitations (Nava et al., 2020). There is a need for much deeper investigations of these processes, which hold potential for the discovery of new therapeutic avenues to improve immune cell infiltration into the TME.

The intricate process of cancer invasion and metastasis involves a dynamic interplay between tumor cells and their surrounding microenvironment. From the initial escape from the primary tumor through the dense and restrictive TME, to the critical steps of intravasation and adaptation to foreign parenchymal environments, cancer cells employ various biochemical and biophysical strategies to survive and propagate. These strategies include nuclear softening, DNA repair, and alterations in the cytoskeletal structure, which enable cells to withstand and respond to mechanical stresses. Furthermore, the role of the cell nucleus in mechanosensing and mechanotransduction has emerged as a pivotal factor, influencing cellular behavior through direct DNA modulation and cytoskeletal interactions. Understanding these complex mechanisms not only highlights the resilience and adaptability of cancer cells but also underscores the challenges faced by anti-tumor immune cells in navigating the confined ECM environment to perform their functions. Continued research into these pathways will be necessary in developing more effective therapeutic targets that can target the unique adaptations of cancer cells heterogenous genetic profiles as they progress throughout the metastatic cascade.

### *In vitro* platforms to investigate the role of compression

To measure and investigate the compressive forces generated during tumor development and progression, various bioengineered platforms have been developed. These platforms provide the tools to analyze the magnitude of intratumoral compressive stress and the effect of compression on malignancy.

#### Tools to quantify compressive stress

To recapitulate accurate models for *in vitro* investigations, the precise magnitudes of mechanical compression in developing tumors must be elucidated. Unlike the characterization of other biomechanical features, the compressive stress exertion in the TME is difficult to directly measure, given its dynamic and 3D context. To date, one of the most prominent methods is to estimate the compressive stress within tumors by applying an incision across the tumor, releasing the stress, and modeling its relaxation to calculate the estimated compressive stress (Levayer, 2020; Stylianopoulos et al., 2012). This method provides a bulk measurement of compression across tumors. Other reports leverage computational modeling of developing tumors, which integrate mechanical features of the tumor, to estimate compressive stress (Bell et al., 2022). From these methods, researchers estimate that compressive stress can range from 500 Pa in glioblastoma to 100–1,000 Pa in colorectal cancer, to 9–10 kPa and 10–42 kPa in pancreatic and breast cancer, respectively. While these approaches are providing new insights into the magnitudes of compression in various cancers, they are still indirectly estimating compression at a single time point. More recently, researchers have developed soft, deformable force probes that can be embedded into biological systems to directly measure mechanical compression with much more resolution and at various time points (Traeber et al., 2019). These deformable force probes are tagged fluorescently, and their deformation profile can be monitored via standard confocal microscopy. Finite element modeling can then be used to extract the compressive stress that corresponds to the deformations of the probe (Traeber et al., 2019; Mohagheghian et al., 2018). As such methods become established, compressive stress development can be accurately quantified in future studies.

#### 2D platforms to assess the role of compressive stress

**FIGURE 2:**
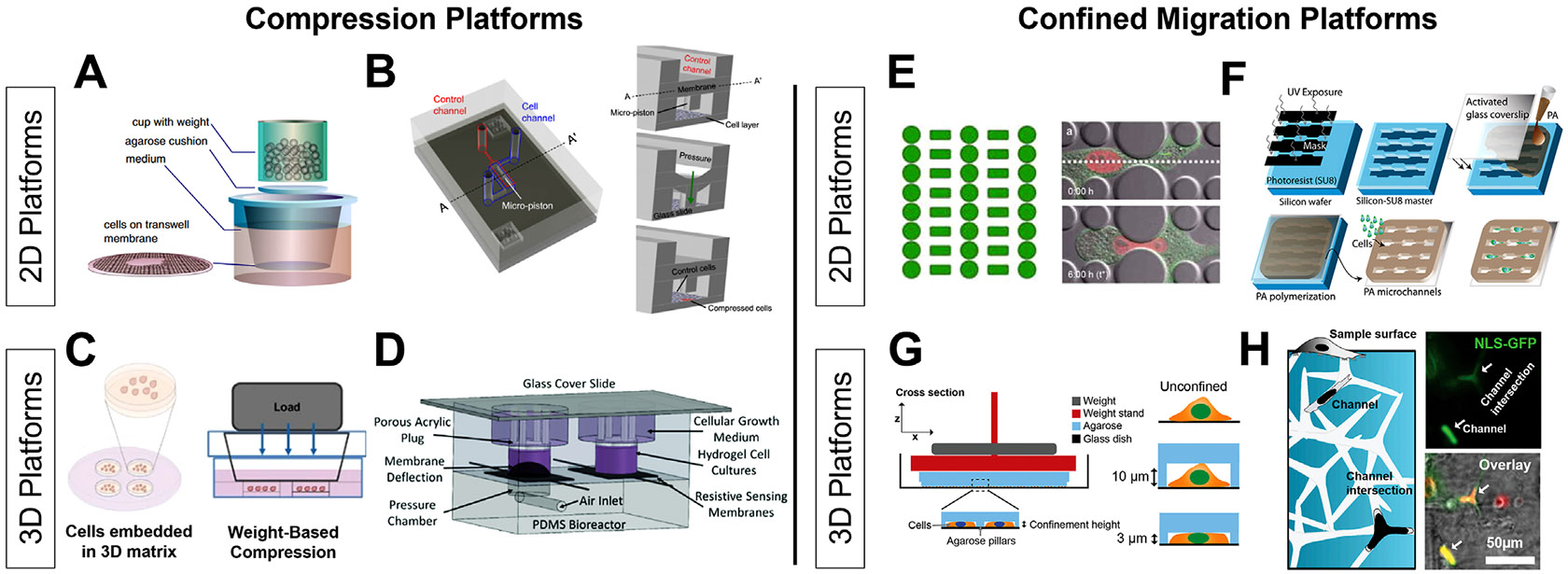
Bioengineering platforms for investigating compressive stress and confined migration 2D platforms leverage transwells and custom-made microfluidic devices to apply compression over a monolayer of cells using weight-based **(A)** and pressure/piston-based approaches **(B)**. 3D application of compressive stress utilizes the encapsulation of cell spheroids in hydrogels before applications of compression through similar weight-based and pressure/piston-based methods **(C, D)**. Various systems create confined environments through designs that simulate cell squeezing **(E)** and confined migration through the use of narrow channels **(F)**. Additionally, confined environments can be generated using tunable substrates such as polyacrylamide **(F)**. Studying confinement in the 3D context have included cells seeded into agarose pillar devices that can apply varying levels of confinement **(G)** and tunable hydrogels with interconnected 3D networks of channels **(H)**. Reproduced with permission A from (Tse et al., 2012), B from (Onal et al., 2021), C from (Kim et al., 2017a), D from (Novak et al., 2020), E from (Davidson et al., 2015), F from (Pathak and Kumar, 2012), G from (Elpers et al., 2023b), H from (Siemsen et al., 2021).

The magnitude at which compressive stress largely depends on the tissue type, as some cancers, like breast cancer, are highly fibrotic and have a high physiologic exposure to compressive stress, while others, like glioblastoma, may have a lower physiologic and pathophysiologic exposure to compressive stress. Existing systems follow similar makeups, consisting of a transwell coupled with an agarose gel and then a loading apparatus (Figure 2A) (Tse et al., 2012; Aureille et al., 2019). The load can be applied through custom made pistons, predefined weights, and pressure-based applications, and applied stress is calculated by determining the amount of force exerted over a defined area. Importantly, this load can be adjusted to recapitulate the magnitude of compression observed within the TME of various cancers, enabling interrogation of compression-dependent mechanisms. The duration of applied compression for each experiment may also differ based on the experimental design, ranging from hours to days. Other systems to study the effects of compression include microfluidic platforms (Figure 2B) and cell-based compression through the use of CAFs surrounding 2D cell cultures (Barbazan et al., 2023) or spheroids (Ermis et al., 2023). Although 2D compressive platforms are unable to fully recapitulate the compressive stress experienced *in vivo*, they allow for precise control of compressive stress and the ease of evaluation of cellular response via established biomolecular assays.

#### 3D platforms to assess the role of compressive stress

The application of multi-axial compressive stress is more indicative of a typical pathophysiologic setting, and therefore produces results better translated to those seen *in vivo*. However, it is much more challenging to analyze compression in the 3D context and platforms for these analyses are still in their infancy. Many existing systems are set up using multicellular aggregates and tumorspheres embedded within a 3D tunable hydrogel that is subject to a compressive load across the hydrogel (Kim et al., 2017b). Similar to 2D designs, these spheroid/gel mixtures are placed within a transwell and load is applied via pistons, weight, or pressure (Figures 2C, D). Current systems only apply a load orthogonal to the hydrogel, and thus create compressive loading distributions that may not resemble those experience *in vivo*. New approaches considering the different loading regimens and applying multi-axial compressive stress are needed. Additional systems can further encapsulate the various systematic changes in response to compressive stress on the vasculature, immune population, matrix reorganization, etc., and allow for further investigation into the role that compressive stress plays in tumor progression. Further testing in 3D could incorporate matrix specifics including integrin specific proteins and crosslinkers that are specific to ECM compositions as seen *in vivo*.

### *In vitro* platforms to investigate confined migration

Studying confinement on both 2D and 3D platforms is important in understanding how cells change their molecular profiles to adjust to the environment that they encounter. For example, invading tumor cells migrate through the dense 3D ECM but also experience trans-endothelial migration and invasion through basement membranes during spread, which can be examined in a more 2D context (Paul et al., 2017). These environments are complex and new and accurate platforms will be key to advance this area of study. This section of the review will detail some of the key platforms in the 2D and 3D space. Through a combination of intravital imaging and tissue ultrastructure analysis, cells are known to traverse pore sizes that are much smaller than the cell size, ranging from 8 μm to even <1 μm. To recapitulate these physical constraints, each of these platforms are designed to present barriers of this size to the cells (Mahaffey et al., 2024; Saif et al., 2023; Alexander et al., 2013).

#### 2D platforms for investigating confined migration

2D platforms for studying confined migration include microfluidic devices and established techniques like transwell migration assays. Microfluidic devices for confined migration typically involve channels with controlled widths or micropillars and mazes that force cells to dramatically deform, simulating confined migration (Figure 2E) (Davidson et al., 2015). The advantages of these platforms are the ease by which they can be imaged and probed via microscopy. Additionally, specific geometries can be imprinted on other hydrogels, giving researchers the ability to encase channels in a matrix with tunable properties, including stiffness. (Figure 2F) (Siemsen et al., 2021). It is also possible to create confinement through coverslips that have a tunable geometric microstructure, and through coating these coverslips with gels of tunable stiffnesses creating an elastically confined environment (Le Berre et al., 2014). Another well characterized 2D confinement assay is the transwell migration and invasion assays. These are advantageous due to the varying pore sizes available that allow for comparisons between several different biologically relevant sizes that cells would squeeze through *in vivo* (Justus et al., 2023). These methods offer valuable insights into cellular mechanics and behavior under confined conditions.

#### 3D platforms for investigating confined migration

In 3D platforms for studying confined migration, 3D tunable hydrogels are a popular choice that can mimic the extracellular matrix and the porosity of the TME, enabling the study of how cancer cells navigate through dense, confined spaces (Geckil et al., 2010). Current methods employ a weight-based confinement, similar to compression platforms, that place cells within agarose-based channels that then are compressed via disks (Figure 2G). Single cells are then subject to microscopic techniques to analyze their response to various degrees of confinement (Elpers et al., 2023a). Recent advances have added more control of the confined paths in 3D hydrogels through the integration of microchannel networks within hydrogels. A recent report leveraged Zn-O tetrapods to form a network template for cells to squeeze through. This template was mixed with a polyacrylamide precursor solution, polymerized, and then the template was hydrolyzed, leaving a network embedded within a 3D polyacrylamide gel (Figure 2H). This system can control for gel stiffness and thus include biophysical parameters important in the TME (Siemsen et al., 2021). With the advancement of *in vivo* imaging techniques, intravital imaging offers real-time visualization of cell behavior within living organisms, providing insights into confined migration within actual tissue environments (Choo et al., 2020). These methods capture the complexity of cancer cell behavior in physiologically relevant, three-dimensional contexts, and will be valuable in elucidating the impact of biophysical cues on the phenotypic and functional changes within tumor and immune cells in confined migration.

### Future perspectives and clinical opportunities

With the interconnected nature of compressive stress in the TME, multiple cell types are continuing experiencing this mechanobiological cue, and future efforts should focus on the integration of multiple cell types into the compression systems. Early reports indicate for instance that compression can initiate fibroblast spreading over cancer cells, recapitulating early organization of cancer (Bertillot et al., 2024b). Creating platforms that include immune cells, stromal cells, and cancer cells can help dissect the role of compression in tumor development and evolution. Engineered co-culture platforms that integrate physiologically relevant compression can also serve as a drug screening technology, lowering the cost of drug development and providing ease for toxicity testing (Brancato et al., 2020).

Mechanistic insights gained from compression/confinement platforms can also be integrated into mechanobiological computational and machine learning models for predicting cancer growth and drug responses (FJBib; Carrasco-Mantis et al., 2023). Mechanobiological models integrate the impact of mechanics on tumor growth, which have been shown to accurately predict tumor dynamics and identify the roles of various mechanobiological factors (Wu et al., 2024). Computational models may also aid in the dissection of the interplay between the emergence of various biophysical factors (i.e., compressive solid stress → ECM secretion → substrate stiffness changes). These insights may enable new generations of *in vitro* platforms that can recapitulate multiple biophysical cues simultaneously. Excitingly, many computational models in this space are pursuing clinically relevant applications by endeavoring to extract patient-specific data for personalized predictions of tumor evolution (Lorenzo et al., 2023; Mascheroni et al., 2021). Future work measuring patient-specific information on tumor mechanobiology will enable integration of these features into personalized models.

Additionally, insights gained from compression/confined migration platforms can also be used to improve existing therapies and identify novel targets for pharmacological intervention. Confined migration platforms can help improve immunotherapies by overcoming the biophysical barriers in the TME. Chimeric antigen receptor (CAR) T-cell therapy and anti-PD-1 therapy rely on T cell infiltration, and as discussed earlier provide a dense biophysical barrier that impedes immune cell invasion. Recent work has shown that halting biophysical TME remodeling and densification by CAFs improved CAR-T cell efficacy (Wang et al., 2014; Sakemura et al., 2022). Reports have also identified that reducing collagen cross-linking through lysyl oxidase inhibition enabled more efficient T cell infiltration into the tumor, leading to improved anti-PD-1 treatment (Nicolas-Boluda et al., 2021). Studies aimed at elucidating the mechanisms T cells use to infiltrate confined spaces may uncover T cell specific pathways that can be leveraged to improve confined migration efficiency. Compression/confinement platforms are also actively used to interrogate the pro-malignant changes that occur in stromal and cancer cells. These efforts have the potential to identify a suite of new pharmacological targets for cancer treatment (Kumari et al., 2023).

As our understanding of the biophysical inputs shaping cancer progression grows, novel avenues of TME therapeutics can be developed. As we detailed above, there are exciting developments and applications in screening platforms, personalized treatment, and immunotherapy. The continued exploration of these important questions will undoubtably yield impactful results.

## Conclusion

The development of compressive stress in early-stage tumor development underlies many of the biophysical changes that emerge during tumor progression. Its sustained impact affects cancer cell proliferation, metabolism, phenotype, and invasion and leads to worse outcomes. Accompanying compression is the formation of the physically dense TME, which forces metastatic cells and infiltrating immune cells to migrate through confined environments. Cancer cells adopt mechanisms to efficiently undergo confined migration, but immune populations struggle to successfully squeeze through submicron sized pores. With the advancement of new bioengineered tools, new insights are being made about how compression is transduced, how compression regulates pro-malignant pathways, and how cells navigate confined environments. The continued investigation of these biophysical inputs will lead to new basic science discoveries and identify novel targets for pharmacological intervention.

## Figures and Tables

**TABLE 1:** Mechanotransduction of biophysical changes in the tumor microenvironment.

Biophysical Changes in TME	Magnitude of Biophysical Cue	Mechanosensitive Receptors and Proteins	Activated Pathways	Refs
Shear Stress	*TME interstitial flow:* 0.1–50 μm/s *shear stress*: 0.007–0.1 dyne/cm^2^	Glycocalyx Primary Cilia Ion Channels Cadherins	MAPK PI3K/Akt EMT programs	Qazi et al. (2013), Yang et al. (2016), Huang et al. (2018a), Mitchell and King (2013), Espina et al. (2023), Tzima et al. (2005), Chivukula et al. (2015)
Tensile Stress	2.2–19 kPa at tumor periphery	Cadherins Piezo TMC Claudins	RhoGTPase FAK-Src YAP/TAZ MRTFs	Charras et al. (2018), Paszek et al. (2005), Conklin et al. (2011), Khilan et al. (2021), Sayyah et al. (2014), Sanz-Moreno et al. (2011), Laklai et al. (2016), Chen et al. (2017), Sun et al. (2016), Karska et al. (2023), Crosas-Molist et al. (2017)
ECM stiffness	4 kPa (Breast) 8–12 kPa (Liver) 6 kPa (Pancreatic) 20–30 kPa (Lung) 1–2 kPa (Glioblastoma) 8 kPa (Bladder)	Integrins DDRs CD44, RHAMM Syndecans	RhoGTPase FAK-Src YAP/TAZ EMT programs	Polacheck et al. (2014), Kim et al. (2019), Kalaitzidou et al. (2024), Berger et al. (2017), Pirentis et al. (2015), Ghasemi et al. (2020), Magazzù and Marcuello (2023), Massey et al. (2024), Wright et al. (2023), Heneberg (2016), Poltavets et al. (2018)
Compressive stress	500 Pa (Glioblastoma) 100-1 kPa (Colon) 9–10 kPa (Pancreatic) 10–42 kPa (Breast)	Vimentin Microtubules Actin → LINC Piezo1	RhoGTPase Src YAP/TAZ EMT programs	Brangwynne et al. (2006), Pogoda et al. (2022), He et al. (2018), Aureille et al. (2019), Luo et al. (2022), Xu et al. (2021b), Monzo et al. (2016), Crosas-Molist et al. (2017)
